# Clinical and x-ray oral evaluation in patients with congenital Zika Virus

**DOI:** 10.1590/1678-7757-2018-0276

**Published:** 2019-05-20

**Authors:** Isabella Fernandes Carvalho, Phillipe Nogueira Barbosa Alencar, Maria Denise Carvalho de Andrade, Paulo Goberlânio de Barros Silva, Ellaine Dóris Fernandes Carvalho, Lavina Sousa Araújo, Michelly Pedrosa Monteiro Cavalcante, Fabrício Bitú Sousa

**Affiliations:** 1 Centro Universitário Christus, Departamento de Odontologia, Fortaleza, Ceará, Brasil.

**Keywords:** Zika virus, Microcephaly, Tooth eruption

## Abstract

**Objective:**

The aim of this study was to investigate possible malformations in the soft, bone and/or dental tissues in patients with congenital Zika Virus (ZIKV) by clinical and x-ray evaluation.

**Methodology:**

Thirty children born with ZIKV and 30 children born without ZIKV (control group) were included in the study. Patients were evaluated over 24 consecutive months according to the variables: sex, age, cleft palates, soft tissue lesions, alveolar ridge hyperplasia, short labial and lingual frenums, inadequate posture of the lingual and perioral muscles at rest, micrognathia, narrow palatine vaults, changes in the teeth shape and/or number, sequence eruption, spasms, seizures and eruption delay were evaluated. Chi-square test, Student's t-test and nominal logistic regression were used (p<0.05).

**Results:**

Among the 30 babies examined, the mean age of the first dental eruption was 10.8±3.8 with almost two-thirds of the children (n=18, 60%) experiencing eruptions of their first tooth after 9 months of age, nine children (30%) had inadequate lingual posture at rest, more than half of the children (n=18, 60%) had short labial or lingual frenums. ZIKV babies showed a high prevalence of clef palate (p<0.001), inadequate lingual posture at rest (p=0.004), micrognathia (p=0.002), changes in the shape and/or number of teeth (p=0.006), alteration in sequence of dental eruption (p<0.001) and muscles spasms (p=0.002). The delay eruption was associated with inadequate lingual posture at rest (p=0.047), micrognathia (p=0.002) and changes in the shape and/or number of teeth (p=0.021). The delayed eruption (p=0.006) and narrow palatine vaults (p=0.008) were independently associated with ZIKV. Moreover, female patients showed the most narrow palatine vaults (p=0.010).

**Conclusions:**

The children with ZIKV showed a greater tendency to have delayed eruption of the first deciduous tooth, inadequate lingual posture and short labial and lingual frenums.

## Introduction

Zika virus (ZIKV), an emerging mosquito-borne flavivirus, was initially isolated from a rhesus monkey in the Zika forest in Uganda in 1947. The virus is transmitted by various species of *Aedes* mosquitoes. In 2015, there was a dramatic increase in reports of ZIKV infection in the Americas. Brazil is the country most affected by this disease, with preliminary estimates of 440,000 to 1.3 million cases of autochthonous ZIKV infection reported through December 2015.^1^

The classic clinical picture of ZIKV infection resembles that of Dengue fever and Chikungunya. The known clinical symptoms are: fever, headache, arthralgia, myalgia, and a maculopapular rash, a complex of symptoms that hinder differential diagnosis.^1^

The evidence of the relationship between ZIKV infection and cerebral birth abnormalities, namely, microcephaly, was first described in January 2015 and has been growing.^2^ The direct cells targeted by ZIKV in the developing human fetus are not clear. Recent studies have shown that a strain of the ZIKV, MR766, which is serially passed from monkey and mosquito cells, efficiently infects human neural progenitor cells (hNPCs) derived from induced pluripotent stem cells.^3^ As the face is formed mainly by the first branchial arch, which is divided into maxillary and mandibular processes, some changes in the oral and craniofacial development can occur, because infections, as syphilis, are contracted by the mother during pregnancy.^4^ ^–^ ^6^

In addition to microcephaly, other changes related to congenital ZIKV infection have been detected in infected children, such as severe ocular lesions, hearing loss, lack of muscle tone and arthrogryposis. For this reason, experts have suggested the creation of the term “congenital syndrome of Zika virus”.^7^ ^,^ ^8^

In January 2017, the epidemiological bulletin from the Ministry of Health showed a national total of 9,770 cases of microcephaly that were reported from March to October 2016. Of these, 2,334 cases were confirmed to be ZIKV infections, while the others remained under investigation or were discarded.^9^ In the state of Ceará, the epidemiological bulletin of the State Secretariat of the Government of the State of Ceará published in May 2017 showed 163 confirmed cases of microcephaly suggestive of congenital ZIKV infection, while the others remained under investigation or were discarded. Fifty-seven confirmed cases were concentrated in the capital of Fortaleza.^10^

Recently, studies showed alteration in the chronology of the first deciduous tooth eruption in Brazilian children with microcephaly associated with ZIKV. Additionally, it has been suggested that these children have great difficulty in dental care due microcephaly.^11^ ^,^ ^12^ So, considering the current panorama that involves a severe spread of ZIKV syndrome, the aim of this study was to investigate possible malformations in the soft, bone and/or dental tissues in patients with congenital ZIKV by clinical and x-ray evaluation.

## Methodology

This study was divided into two phases: an initial cross-sectional observational study that included 30 children born with congenital ZIKV and microcephaly; and a case-control study in which more 30 control children born without congenital ZIKV were analyzed for comparison. The microcephaly diagnosis was based on the guideline of the Brazilian Ministry of Health, which defines microcephaly babies as the newborns with 37 weeks or more of gestational age and cephalic perimeter ≤31.5 cm for girls and ≤31.9 cm for boys.^13^

All children were referred from medical services to the School of Dentistry Centro Universitário Christus (Unichristus) department of dental care for patients with special needs. A convenience sample of 30 children with ZIKV was evaluated. After the informed consent form was obtained, the children were examined and followed up over 24 consecutive months. The inclusion criteria were children with ZIKV and microcephaly, born full term, and with availability for oral evaluations every three months during the follow-up time. Children with microcephaly due to other causes were excluded. Data collection began in April 2016 and ended in April 2018.

After anamnesis and verification of past medical history, a clinical examination was performed. Possible soft and hard tissue changes and dental development, chronology of eruption and possible malformations, were observed.

**Figure 1:**
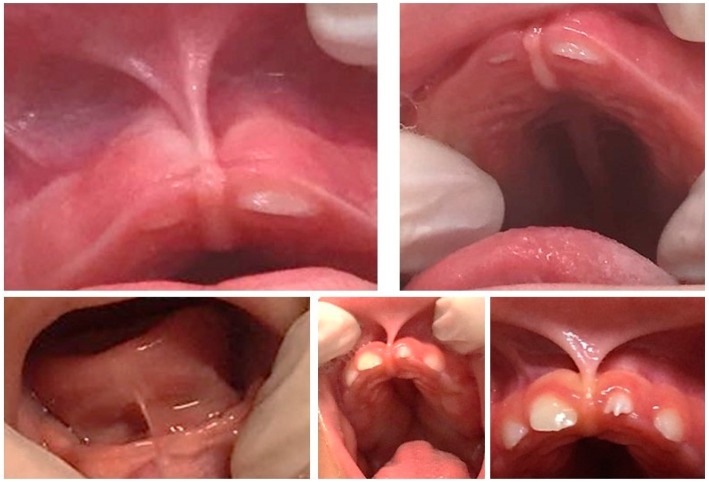
Intraoral profile of babies with congenital ZIKV infection exhibiting shortening of upper and lower lingual frenums

**Figure 2:**
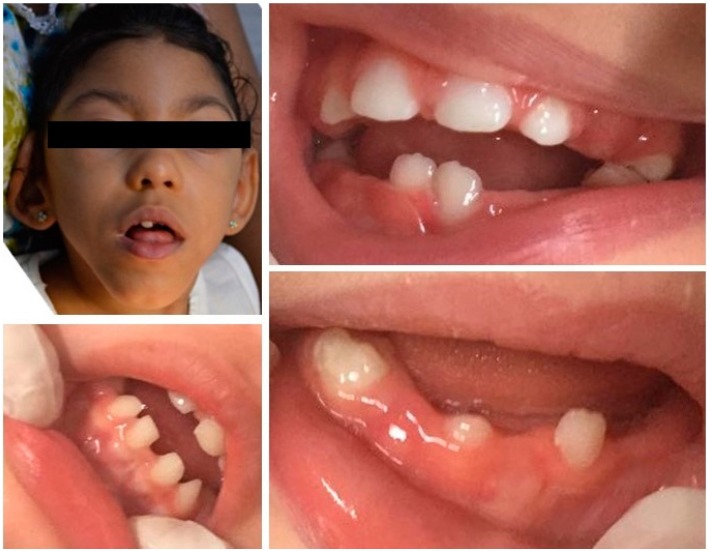
Orofacial and dental profile in babies with congenital ZIKV infection exhibiting narrow palatine vaults and dental eruption interference

First, an extrabuccal examination of the cervicofacial region [palpation of the cervicofacial lymph nodes, (subject position: child sat on the parents’ lap in front of the examiner)] and parotid glands examination (subject position: child lying on a dental chair with head tilted slightly backwards) were performed. During the intraoral examination, the child remained lying on the parents’ lap for visual inspection and bidigital palpation. Since the neurological impairments caused by microcephaly unbalance the lingual posture, this outcome was assessed when the child was in a resting position^14^ (Figures 1 and 2).

In babies with congenital ZIKV infection, radiographs were used to diagnose possible dental anomalies in the number, shape or position of teeth and to visualize the extent of probable bone-alveolar defects in the patients. A modified periapical technique was performed with an adult phosphor plate (number 2) in an occlusal position, in an attempt to visualize a larger amount of the dental elements and adjacent bones in both arches. The long axis of the film was positioned in the oral cavity; perpendicular to the median sagittal plane and stabilized with the labial commissures, with the parents’ assistance.^15^ This modification technique was adopted as an easy method that requires less child cooperation. Two radiographs were performed *per* child, one for the maxillary arch and another for the mandible arch.

For periapical radiographs of the maxillary arch, the central beam of radiation was directed to the glabellar region with a horizontal angulation of 0 degrees and a vertical angulation of 65 degrees. For the mandibular arch, the central beam was directed to the median region of the buccal floor with a horizontal angulation of 0 degrees and a vertical angulation perpendicular to the long axis of the plaque. Patients were placed on the parents’ lap, using all the necessary protection barriers to perform the tests properly.^15^

The use of digital images is known to be more beneficial than the use of conventional images, both for exam operators and for patients. Given that digital receivers are more sensitive to radiation, a lower dose of radiation should be used to reduce the risk of possible deleterious effects of radiation for patients. The ease of storage of the images and the ability to alter the brightness and contrast to improve the images and diagnosis are also important factors;^16^ in this context, the device used was the Express digital imaging plate scanner (Express, Instrumentarium Dental Inc., Willwaukee, Wisconsin, USA). The exposure time for each x-ray outlet was 0.25 s. After image acquisition and processing, the Cliniview software was used for image interpretation and brightness and contrast adjustments as necessary.

In this study, a delay in the first dental eruption was considered if children were 9 months old or older at the time of the first eruption. These data were based on studies about the mean ages of eruption: for the lower central incisors the mean age of eruption was between 7 and 9 months; for the lower lateral incisors it was between 12 and 14 months; for the upper central incisors it was between 9 and 11 months; and for the upper lateral incisors it was between 10 and 12 months.^17^ ^–^ ^19^

Categorical data were expressed as the absolute and percent frequency and compared using the Pearson's chi-square test, and the quantitative data were expressed as the mean and standard deviation and compared with Student's *t*-test. Multinomial Logistic Regression was used in variables that showed significant association in the case control phase of the study from adjust clinic factors that can interfere in oral and systemic outcomes. All analyses were performed with the software Statistical Package for the Social Sciences version 17.0 for Windows, adopting a 95% confidence level.

The study was approved by the Ethics Committee of the educational institution of the participants, according to the protocol CEP: 60740616.4.0000.50.49. A free and informed consent form was read and signed by the parents of the participants before the research was performed.

## Results

### Results of the cross-sectional observational study

Among the patients evaluated, 14 (46.7%) were female and 16 (53.3%) were male. At the end of the study, 18 children (60%) were less than 25 months old and 12 children (40%) were 25 months old or older.

**Figure 3:**
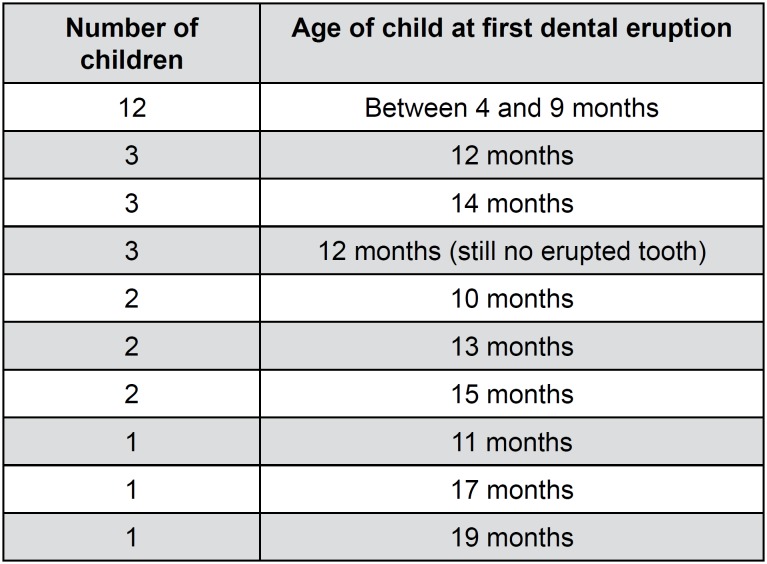
Results for age at first dental eruption and number of children

The mean age of the first dental eruption of children in this study was 10.8±3.8 months, with almost two-thirds of the children (n=18, 60%) experiencing eruptions of their first tooth after 9 months of age. In all cases, the first eruptions were of the lower central incisors (Figure 3 and Table 1).

**Table 1:** Evaluation of clinical characteristics and delays in the first eruptions of ZIKV children

		Eruption delay			Sex		
	Total	No	Yes	p-value	Female	Male	p-value
**Sex**							
Female	14	7	7	0.457	–	–	–
	46.7%	58.3%	38.9%		–	–	
Male	16	5	11		–	–	
	53.3%	41.7%	61.1%		–	–	
**Age**							
Less than 25 months	18	9	9	0.171	9	9	0.722
	60.0%	75.0%	50.0%		64.3%	56.3%	
25 months or more	12	3	9		5	7	
	40.0%	25.0%	50.0%		35.7%	43.8%	
**Hyperplasia of the alveolar ridge**							
No	29	12	17	1.000	13	16	0.467
	96.7%	100.0%	94.4%		92.9%	100.0%	
Yes	1	0	1		1	0	
	3.3%	0.0%	5.6%		7.1%	0.0%	
**Short labial or lingual frenum**							
No	12	6	6	0.361	6	6	0.765
	40.0%	50.0%	33.3%		42.9%	37.5%	
Yes	18	6	12		8	10	
	60.0%	50.0%	66.7%		57.1%	62.5%	
**Inadequate lingual posture at rest**							
No	21	11*	10	0.047	11	10	0.440
	70.0%	91.7%	55.6%		78.6%	62.5%	
Yes	9	1	8*		3	6	
	30.0%	8.3%	44.4%		21.4%	37.5%	
**Micrognathia**							
No	28	12	16	0.503	14	14	0.485
	93.3%	100.0%	88.9%		100.0%	87.5%	
Yes	2	0	2		0	2	
	6.7%	0.0%	11.1%		0.0%	12.5%	
**Narrow palatine vaults**							
No	20	6	14	0.114	6	14*	0.010
	66.7%	50.0%	77.8%		42.9%	87.5%	
Yes	10	6	4		8*	2	
	33.3%	50.0%	22.2%		57.1%	12.5%	
**Changes in the shape and/or number of teeth**							
No	26	12	14	0.130	13	13	0.602
	86.7%	100.0%	77.8%		92.9%	81.3%	
Yes	4	0	4		1	3	
	13.3%	0.0%	22.2%		7.1%	18.8%	
**Sequence of dental eruption**							
No	26	11	15	0.632	4	9	0.159
	86.7%	91.7%	83.3%		28.6%	56.3%	
Yes	4	1	3		10	7	
	13.3%	8.3%	16.7%		71.4%	43.8%	
**Spasms**							
No	13	5	8	1.000	8	13	0.236
	43.3%	41.7%	44.4%		57.1%	81.3%	
Yes	17	7	10		6	3	
	56.7%	58.3%	55.6%		42.9%	18.8%	
**Seizures**							
No	21	7	14	0.418	11	14	0.642
	70.0%	58.3%	77.8%		78.6%	87.5%	
Yes	9	5	4		3	2	
	30.0%	41.7%	22.2%		21.4%	12.5%	
**Others**							
No	25	8	17	0.128	13	13	0.602
	83.3%	66.7%	94.4%		92.9%	81.3%	
Yes	5	4	1		1	3	
	16.7%	33.3%	5.6%		7.1%	18.8%	

* p<0.05, Fisher's exact test or Pearson's Chi-square test

In addition, the last child to present an eruption was 19 months of age. Moreover, three children who were 12 months old had no teeth in their oral cavities at the end of the study. Twelve children in the sample had their first eruptions at up to 9 months of age (Figure 3).

No patients had cleft palates or soft tissue lesions. Only one child (3.3%) had hyperplasia of the alveolar ridge. More than half of the children (n=18, 60%) had short labial or lingual frenums. Nine children (30%) had inadequate lingual posture at rest, and two children (6.7%) had micrognathia. Ten children (33.3%) had narrow palatine vaults. Four children (13.3%) had changes in the shape and/or number of teeth. Three patients showed agenesis of deciduous/permanent lower and upper incisors and one patient presented microdontia associated to accessory cusps (left upper central incisor).

Four children (13.3%) had an altered sequence of dental eruption. Twelve children (56.7%) presented spasms, nine (30%) had seizures, and five (16.7%) had other comorbidities such as severe hearing and visual problems (Figure 3).

In terms of delays in the first dental eruption, there was only a significant association in patients with inadequate lingual posture at rest. Nine children had altered postures and eight of these had their first teeth eruptions delayed (Figure 3 and Table 1). The frequency of inadequate posture was 7.3 (95% CI=1.1 – 68.9) times higher in patients with delayed dental eruptions than in patients who had no delayed eruptions.

Changes in the dental eruption sequence were found in 4 children, in whom the first deciduous molars erupted before all the lower incisors were present in the oral cavity.

In this study, micrognathia was associated with delays in the first dental eruption. An association 38% higher of micrognathia was observed in patients who presented delay on first dental eruption when compared with patients without delay. In addition, patients with poor dental formation had delays in the first dental eruption that were on average 20% higher compared to patients without this condition. There was no statistically significant difference between the mean time to the first dental eruption and the other variables. Female patients had a high prevalence of narrow palatine vaults when compared to male patients (p=0.010). The other variables showed no association with sex (Table 1).

Patients with short labial and/or lingual frenums were referred to a department specialized in pediatric dentistry to have a frenectomy or a frenotomy performed, when indicated.

### Results of the case-control study

A total of 30 babies born without ZIKV were used for comparison of clinical characteristics and calculation of the influence of ZIKV in prevalence of dental alterations. There is no statistical difference between sex distribution in the two groups (p=1.000) and most children were boys in both groups. At the end of the data collection, the age of control group was significantly lower than the ZIKV group (p=0.015).

**Figure 4:**
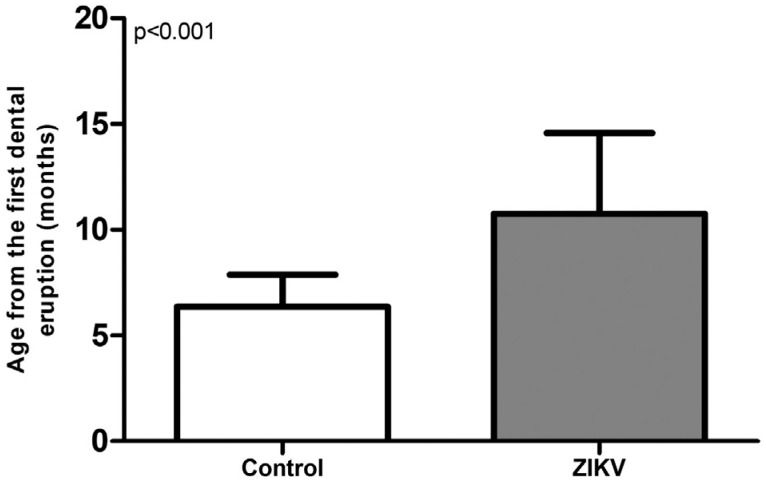
Age at which the first tooth erupted in ZIKV and control group *Student's t-test (mean±SD)

The age at which the first dental eruption occurred was significantly later in the ZIKV group (10.8±0.7 months) compared to the control group (6.3±0.3 months) (p<0.001) (Figure 4). The prevalence of delayed eruption was 43.5 (CI 95%=5.2 – 363.7) times higher in ZIKV group than in control group (n=1, 3.3%) (p<0.001). No patients had cleft palates or soft tissue lesions, similar to the control group (p=1.000); and no children in control group showed hyperplasia of the alveolar ridge, similar to the ZIKV group in this parameter (p=1.000) (Table 2).

**Table 2:** Evaluation of clinical characteristics and eruption time in ZIKV children

	Age of the first dental eruption (months)	p-value
**ZIKV group**	10.8±3.8	–
**Sex**		
Female	9.9±4.3	0.266
Male	11.5±3.3	
**Age**		
Less than 25 months	10.9±4.3	0.444
25 months or more	10.5±3.1	
**Hyperplasia of the alveolar ridge**		
No	10.7±3.9	0.748
Yes	12.0±0.0	
**Short labial or lingual frenum**		
No	9.4±3.5	0.114
Yes	11.7±3.8	
**Inadequate posture at rest**		
No	9.9±3.6	0.028
Yes	12.8±3.6*	
**Micrognathia**		
No	10.5±2.8	0.002
Yes	14.5±0.7*	
**Narrow palatine vaults**		
No	11.2±3.5	0.334
Yes	9.8±4.4	
**Changes in the shape and/or number of teeth**		
No	10.5±4.0	0.021
Yes	12.7±1.0*	
**Sequence of dental eruption**		
No	10.7±4.0	0.79
Yes	11.2±3.0	
**Seizures**		
No	11.5±3.7	0.096
Yes	9.0±3.6	
**Others**		
No	11.4±3.8	0.054
Yes	7.8±2.9	

* p<0.05, Student's t-test

Short labial or lingual frenums were 4.9 (CI 95%=1.6 – 15.1) times higher in ZIKV group than control group (p=0.004), inadequate lingual posture at rest showed a prevalence 26.9 (CI 95%=1.5 – 488.7) times higher in ZIKV children (p=0.002) and micrognathia did not differ for control group (p=0.492). Narrow palatine vaults was 14.5 (CI 95%=1.7 – 122.5) times higher in babies with congenital ZIKV infection and changes in the shape and/or number of teeth showed no difference compared to control group (p=0.112) (Table 2).

Spasms and seizures were significantly most frequent in babies with congenital ZIKV infection with a prevalence 79.1 (CI 95%=4.4. – 1414.0) and 26.9 (CI 95%=1.5 – 488.7) higher than control group (p<0.001 and p=0.003, respectively). Other comorbidities such as severe hearing and visual problems did not differ from control group (p=0.052) (Table 2).

### Results of the multivariate analysis

In multivariate analysis (adjustment by age) the factors most prevalent in babies with congenital ZIKV infection were delayed eruption (p=0.006) and narrow palatine vaults (p=0.008). The adjusted odds ratios were 26.6 (CI 95%=2.5 – 279.5) and 24.8 (CI 95%=2.3 – 270.1) higher than in control group, respectively. The systemic alterations showed no significant differences in ZIKV and control groups when adjusted by age (Table 3). Therefore, age is a determinant factor of systemic alterations, development of short labial or lingual frenums and inadequate lingual posture at rest while delayed eruption and narrow palatine vaults are independently most prevalent in babies with congenital ZIKV infection (Table 4).

**Table 3:** Case-control evaluation of clinical characteristics and delays in ZIKV children

	Group		
	Control	ZIKV	p-value
**Sex**			
Female	13	14	1.000
	43.3%	46.7%	
Male	17	16	
	56.7%	53.3%	
**Age**			
Less than 25 months	27*	18	0.015
	90.0%	60.0%	
25 months or more	3	12*	
	10.0%	40.0%	
**Delayed tooth eruption**			
No	29*	12	<0.001
	96.7%	40.0%	
Yes	1	18*	
	3.3%	60.0%	
**Hyperplasia of the alveolar ridge**			
No	30	29	1.000
	100.0%	96.7%	
Yes	0	1	
	.0%	3.3%	
**Short labial or lingual frenum**			
No	23*	12	0.004
	76.7%	40.0%	
Yes	7	18*	
	23.3%	60.0%	
**Inadequate lingual posture at rest**			
No	30*	21	0.002
	100.0%	70.0%	
Yes	0	9*	
	.0%	30.0%	
**Micrognathia**			
No	30	28	0.492
	100.0%	93.3%	
Yes	0	2	
	.0%	6.7%	
**Narrow palatine vaults**			
No	29*	20	0.006
	96.7%	66.7%	
Yes	1	10*	
	3.3%	33.3%	
**Alteration in sequence of dental eruption**			
No	30	26	0.112
	100.0%	86.7%	
Yes	0	4	
	0.0%	13.3%	
**Spasms**			
No	30	13	<0.001
	100.0%	43.3%	
Yes	0	17	
	.0%	56.7%	
**Seizures**			
No	30	21	0.003
	100.0%	70.0%	
Yes	0	9	
	0.0%	30.0%	
**Others**			
No	30	25	0.052
	100.0%	83.3%	
Yes	0	5	
	.0%	16.7%	

* p<0.05, Fisher's exact test or Pearson's Chi-square test

**Table 4:** Multivariate analysis evaluating the influence of age on the prevalence of oral and systemic alterations in infants with ZIKV

	p-value	Adjusted OR (CI 95%)
**ZIKV group**		
**Oral alterations prevalence adjusted from age**		
Age	0.118	–
Delayed eruption	0.006	26.6 (2.5 – 279.5)
Short labial or lingual frenum	0.239	–
Inadequate lingual posture at rest	1.000	–
Narrow palatine vaults	0.008	24.8 (2.3 – 270.1)
**Systemic alterations prevalence adjusted from age**		
Age	0.181	–
Spasms	0.995	–
Seizures	1.000	–

* p<0.05, Multinomial Logistic Regression test. Adjusted OR = Adjusted Odds Ratio from age; CI 95% = 95% Confidence Interval of Adjusted OR

## Discussion

This is the first two-phase study on ZIKV that consists of a cross-sectional observational study and a case control study that evaluated oral and tooth changes in children with ZIKV and microcephaly. The major limitation of this study is the difficulty of recruiting large numbers of patients, since the total number of patients in the city of Fortaleza is low (N=56). In addition, these patients present a great difficulty in accessing the health service, as well as difficulty during examinations and x-ray evaluation due to neurological impairment. However, this study showed some relevant initial contributions about the development of oral structures in babies with ZIKV.

The existing Brazilian literature, in agreement with the world literature, indicates that the average age of the first dental eruption is 9 months, and a delay in eruption is considered after this period.^17^ In this study, considering congenital ZIKV children, approximately two-thirds of the patients (n=18, 60%) had eruptions of the first tooth after 9 months of age. In the control group, some patients had considerable dental delays, with eruptions at 15, 17 and 19 months.

The most significant result in this study was the delay in tooth eruption, which was 26.6 times more prevalent in ZIKV group than in control group, with a significantly higher average for the first eruption. The suggestion of this study is that delays in dental eruption are associated with congenital ZIKV syndrome. One of the hypotheses for why delayed tooth eruption occurs is due to failure of the precise mechanisms of dentition development resulting from the infection of human neural progenitor cells by the ZIKV, which culminates in microcephaly and may affect the physiological processes for eruption.^4^ ^,^ ^6^

The narrow palatine vault was 24.8 times more prevalent in ZIKV group than control group, affecting preferably female children. Additionally, the inadequate lingual posture at rest was more frequent in ZIKV children with delayed tooth eruption. The narrow palatine vault may result in an inadequate oral posture due hypotonia of orofacial musculature. The ZIKV infects human neural progenitor cells (hNPCs) derived from induced pluripotent stem cells leading to neurological alterations.^3^ It is suggested that neurological alterations lead to hypotonia of orofacial musculature, followed by inadequate tongue posturing, and resulting in poor swallow reflex, and frequent mouth breathing.^20^ ^,^ ^21^ When the mouth is left ajar for long periods, there is limitation of the adequate transverse growth of the maxilla, leading to a tendency toward maxillary atresia and an ogival palate.^22^ ^,^ ^23^

In terms of the existence of other abnormalities, an inappropriate lingual posture at rest was observed in 9 of the 30 ZIKV children and was significantly more prevalent than in control group. Of these 9 children, 8 also had delayed dental eruptions (p=0.047) (Figure 3). This finding may be related to the presence of a more severe neurological condition, such as microcephaly; thus, in addition to an inadequate lingual posture at rest, a delay in the first dental eruption occurs. The structures of the face are mainly composed of cells derived from the neural crest of the embryo.^6^ ^,^ ^20^ ^,^ ^21^

More than half of the children had short labial or lingual frenums with a prevalence 4.9 times higher in ZIKV group than control group. However, when those variables were adjusted by age in multivariate analysis, the association was not significant. The prevalence of short labial or lingual frenums can occur in newborns, but it is has a significant lower occurrence in older children.^24^ Because this study assessed ZIKV patients for a short period of time, the results cannot guarantee that these soft tissue abnormalities will persist throughout the years.

Although there was no significant difference between the control group, 4 children after clinical and x-ray evaluations showed alterations in the shape and/or number of teeth or change in the sequence of tooth eruption. Such occurrences may also be linked to ZIKV infection of cells that cause the development of dental tissues, thus affecting the correct development of the teeth, both in shape and in number.^4^ ^,^ ^6^

Additionally, considering the other characteristics examined, such as presence/absence of cleft lip/palate, soft tissue lesions, alveolar ridge hyperplasia and micrognathia, no conclusive findings related to ZIKV infection were observed.

Considering that the virus infects human neural progenitor cells and that these same cells are responsible for migrating and forming the oral and facial structures during embryogenesis,^4^ further complications may occur late in the course of disease; thus, children will be followed up for medium- and long-term periods to monitor the growth and development of their oral structures. Thus, in the future, a complete skull and facial growth and development profile related to congenital ZIKV syndrome will be proposed.

## Conclusions

The results obtained in this study demonstrate that children with congenital ZIKV syndrome showed a greater tendency to have delayed eruption of their first deciduous tooth, inadequate posture of the lingual, narrow palatine vaults and short labial and lingual frenums. Although the findings are preliminary, the presence of dental changes in the number and shape of teeth and alterations in the eruption sequence are another important finding of this study.
